# Editorial: From individual to collective: bridging the gap between clinical practice and public policies

**DOI:** 10.3389/fpsyg.2023.1169159

**Published:** 2023-05-10

**Authors:** Francisco José Eiroa-Orosa, Gillian MacIntyre

**Affiliations:** ^1^Section of Personality, Evaluation and Psychological Treatment, Department of Clinical Psychology and Psychobiology, School of Psychology, University of Barcelona, Barcelona, Catalonia, Spain; ^2^School of Social Work and Social Policy, University of Strathclyde, Glasgow, United Kingdom

**Keywords:** citizenship, clinical practice, mental health policy, public mental health, recovery

The true integration between different ecological levels (individual practitioner, organizational, regulatory and social levels) of mental health practice evidence remains an ongoing task and there remains an implementation gap between mental health policy and practice (Raghavan et al., 2008; Collins et al., 2013; Bruns et al., 2019). While much scientific literature continues to be produced on the efficacy and effectiveness of interventions there has been limited consideration of the challenges of implementation at a practice level. Indeed, research on public mental health policies can, at times, overlook the idiosyncrasies and needs of practitioners and service users (Green, 2009; Proctor et al., 2009). At a methodological level, the internal validity of research has been traditionally prioritized over clinical or lived experience (Willemsen, 2022). Meanwhile, there are growing voices from the social sciences encouraging analysis of the socio-historical aspects that have led to current mental health approaches (Cosgrove and Karter, 2018) to propose alternative types of research, knowledge and practice (e.g., Johnstone and Boyle, 2018). These differing research paradigms coexist within a scenario of growing demands, at both the level of care pressure and the appearance of new problems and fields of intervention. This scenario leads to practitioners disengaging from the system, as they do not feel that public policies are intended to aid their work and do not adequately reflect the reality and challenges of their practice experiences. Meanwhile, public administrators experience frustration as they struggle to implement their reforms, resulting in tension between both parties (Glasby and Tew, 2015).

In the process of editing this Research Topic, the world witnessed the outbreak of the biggest pandemic in a century. Apart from the immediate consequences of the confinement periods, the pandemic has had a clear impact on the mental health of the population and on its management by public administrations (Torales et al., 2020; Xiong et al., 2020; Kumar and Nayar, 2021). These challenges, combined with a global cost of living crisis (Keith Neal, 2022; Broadbent et al., 2023) have significant public health implications that will put existing health and social care services, staff and service users under significant pressure (Cogan et al., 2022a).

In this Research Topic, we have tried to integrate topics usually addressed in the public mental health literature such as fairness, social justice, empowerment, and participation with those more typically researched at the micro levels such as clinical effectiveness and its covariates or subjective experiences of service users and practitioners. Taking an ecological approach, we have tried to integrate all levels of evidence in a framework that thoroughly includes the social context, and the approach to service users as experts on their own lives, moving away from top-down to bottom-up approaches. In doing so we acknowledge that mental health policy and practice forms part of a complex system (Rutter et al., 2017). This conceptualization involves several interdependent elements that form a connected whole demanding a range of different methods to design, implement and evaluate interventions.

The approach that we adopt in our own work explores the relationship between citizenship and mental health and we suggest that it offers a useful lens through which to understand the multi-layered complex systems that impacts on an individual's mental health and their ability to engage meaningfully as citizens in their local communities. Changes at multiple levels of the complex system may be required to promote better outcomes for individuals. Indeed, what unites the editors of this special issue is our membership of the International Recovery and Citizenship Collective (IRCC), that promotes a framework of understanding of mental health and social inclusion that pays particular attention to the socio-political and cultural context of people with lived experience of mental health difficulties. The concept of Citizenship (Rowe et al., 2001) has been used as a framework (Atterbury and Rowe, 2017) for fostering social participation among members of stigmatized groups. Interventions carried out under this framework, view participants as “citizens” rather than problems to be addressed through the intervention of others (Rowe, 2015). This approach considers participants as experts on their experiences, identifying solutions and taking actions to become valued members of their communities. Accordingly, intervention proposals formulated within the citizenship framework understand that success should not be based simply on symptom alleviation. On the contrary, citizenship focused interventions are based on the idea that persons who enjoy their mental health are persons who use their rights and respect those of others; take responsibilities considering the risks involved; undertake socially valued roles considering both their preferences and needs as well as those of other members of their communities; have access to the resources they need to promote their mental health and wellbeing (such as health services and education); and establish relationships of mutual support and reciprocity (Eiroa-Orosa, 2019, 2023).

Citizenship-based programmes involving people with different psychosocial needs have been developed internationally in different sociocultural contexts (Pelletier et al., 2013; Eiroa-Orosa and Rowe, 2017; Hamer et al., 2019; MacIntyre et al., 2019), and the need to measure the effect of such interventions has emerged. Hence, participatory action research methods in partnership with peer researchers have been employed to develop a measure of Citizenship in the United States (Rowe et al., 2012; O'Connell et al., 2017). Recently, the participatory process has been replicated in Scotland (Cogan et al., 2022b; MacIntyre et al., 2022) and is now underway in Spain.

Regarding our Research Topic, we have included seven works that focus their efforts at different ecological levels. Regarding analyses at the micro level, Drivenes et al. illustrated the struggle in mental health care to establish a common understanding between service users and therapists in decisional processes regarding treatments.

Works framed at the meso-level include the work of Cases et al., who recount the creation and validation of a method to examine discrepancies between guidelines relating to persons diagnosed with borderline personality disorder and current clinical practices in psychiatric emergencies. In a more practical vein, Havsteen-Franklin et al. developed a program logic model for arts-based psychosocial practice within South African rural communities.

At the macro level, Su et al. report the methodology of a large-scale mental health survey carried out in the 85 million-inhabitants Chinese Sichuan province. Guo et al. conducted a meta-analysis of the mental health literacy levels of medical staff in China finding lower literacy rates in developing regions. Li and Zhang used a large data sample of insurance reimbursement settlements to estimate the effect of a payment reform on the quality of public healthcare. Finally, Li et al. also analyzed the impact of family doctor contracting on medical expenses.

We believe that the works included in this Research Topic contribute to promoting critical thinking in public health at different levels of the complex system of healthcare, from daily practice to public policies, thereby embodying a citizenship focused approach to mental health. Perhaps the difficulties encountered by humanity in recent years have also prevented us from collecting a broader set of works with similar perspectives on mental health. However, we hope that these works provide alternative views to their readers, helping to promote an integrated view of mental health care.

## Author contributions

All authors listed have made a substantial, direct, and intellectual contribution to the work and approved it for publication.
